# Elevated cerebrovascular resistance index is associated with cognitive dysfunction in the very-old

**DOI:** 10.1186/s13195-014-0080-3

**Published:** 2015-01-21

**Authors:** Lindsay R Clark, Daniel A Nation, Christina E Wierenga, Katherine J Bangen, Sheena I Dev, David D Shin, Lisa Delano-Wood, Thomas T Liu, Robert A Rissman, Mark W Bondi

**Affiliations:** San Diego State University/University of California, San Diego Joint Doctoral Program in Clinical Psychology, San Diego, CA USA; Department of Psychology, University of Southern California, Los Angeles, CA USA; Department of Psychiatry, University of California San Diego, San Diego, CA USA; Department of Veterans Affairs, San Diego Healthcare System, San Diego, CA USA; Center for Functional MRI, University of California San Diego, San Diego, CA USA; Department of Neurosciences, University of California San Diego, San Diego, CA USA; Wisconsin Alzheimer’s Institute, School of Medicine and Public Health, University of Wisconsin-Madison, Madison, WI USA

## Abstract

**Introduction:**

Age-related vascular changes, including blood pressure elevation and cerebral blood flow (CBF) reduction, are associated with cognitive decline and Alzheimer’s disease (AD). Evidence suggests that the relationship between blood pressure and dementia risk varies between younger and older samples within the elderly population.

**Methods:**

We examined the relationship between mean arterial pressure (MAP), CBF, and cognition in young-old (60 to 75 years of age) versus very-old (80+ years of age) adults. Fifty-eight non-demented older adults completed an arterial spin labeling MRI scan, and an index of cerebrovascular resistance (CVRi) was estimated for each participant by calculating the ratio of MAP and CBF.

**Results:**

Results demonstrated a similar negative relationship between MAP and CBF across both age groups. However, very-old participants exhibited elevated CVRi and reduced CBF compared to young-old participants in regions implicated in AD and cerebral small vessel disease. Furthermore, significant age by CVRi interactions revealed that elevated CVRi in the thalamus was inversely related to verbal fluency performance in the very-old group.

**Conclusions:**

Findings support CVRi as a potential vascular biomarker and suggest that regionally-specific vascular changes may contribute to cognitive decline, particularly in the very-old.

## Introduction

Growing evidence suggests that cerebral small vessel disease is more common and severe in adults over the age of 80 (termed the ‘very-old’) and that elevated cerebrovascular disease burden may lower the threshold of Alzheimer’s disease (AD) pathology necessary to produce clinical signs of dementia [1,2]. Additionally, studies have reported a greater prevalence of mixed AD and vascular pathology in the oldest-old population [3]. Thus, investigating markers of cerebrovascular disease in individuals over the age of 80 is particularly important, as this group is both the fastest growing segment of the population and at highest risk for dementia [4].

A dynamic, non-linear relationship exists between arterial blood pressure (BP) and risk for AD and stroke with advancing age [5]. Studies have consistently documented the association between midlife hypertension and increased risk for AD [6]; however, the relationship between BP and dementia in later life is less clear, with some studies suggesting that lower BP infers greater risk for dementia in advanced age. Furthermore, subtle cerebrovascular dysfunction associated with BP elevation may lead to reduced cerebral blood flow (CBF) and contribute to risk for cerebrovascular disease [7]. Although widespread reduction of CBF occurs during normal aging [8-10], the relationship between CBF and BP may influence the development of cerebrovascular disease and cortical atrophy, promoting vulnerability to neurodegenerative disease [11].

Cerebral autoregulation serves to maintain a constant level of global CBF (for example, typically around 50 ml/100 g/minute) in the context of spontaneous fluctuations in mean arterial pressure (MAP) across a broad physiologic range (for example, between 60 and 150 mmHg) [12]. The relationship between MAP and CBF largely defines the degree of cerebrovascular resistance in the presence of normal intracranial pressure (ICP). Cerebrovascular resistance may be expressed as the ratio of cerebral perfusion pressure (P_*α*_) to CBF (P_*α*_/CBF), where P_α_ = MAP – ICP [13]. To maintain constant CBF, vessels constrict during hypertension and dilate during hypotension [14], and cerebrovascular resistance typically remains low to sustain the high blood supply needed to the brain [15]. Recent studies indicate that cerebral autoregulation remains relatively preserved during normal aging and in AD at a steady state [16]. However, during aging, the lower end of the autoregulatory curve shifts towards higher BP, which may increase sensitivity of the brain to hypoperfusion at lower BPs [12,17], and cerebral autoregulation mechanisms may be disrupted in the context of chronic hypertension and fluctuations in BP [18,19]. Despite the well-established association between elevated BP and reduced CBF with age, few studies have examined the interaction between BP and subtle, regional microvascular CBF at a steady state in the very-old. Greater understanding of the relationship between BP, CBF and cognition in very-old cohorts may improve identification of individuals at risk for cognitive decline and advance the development of treatments to slow the rate of decline.

**Figure 1:**
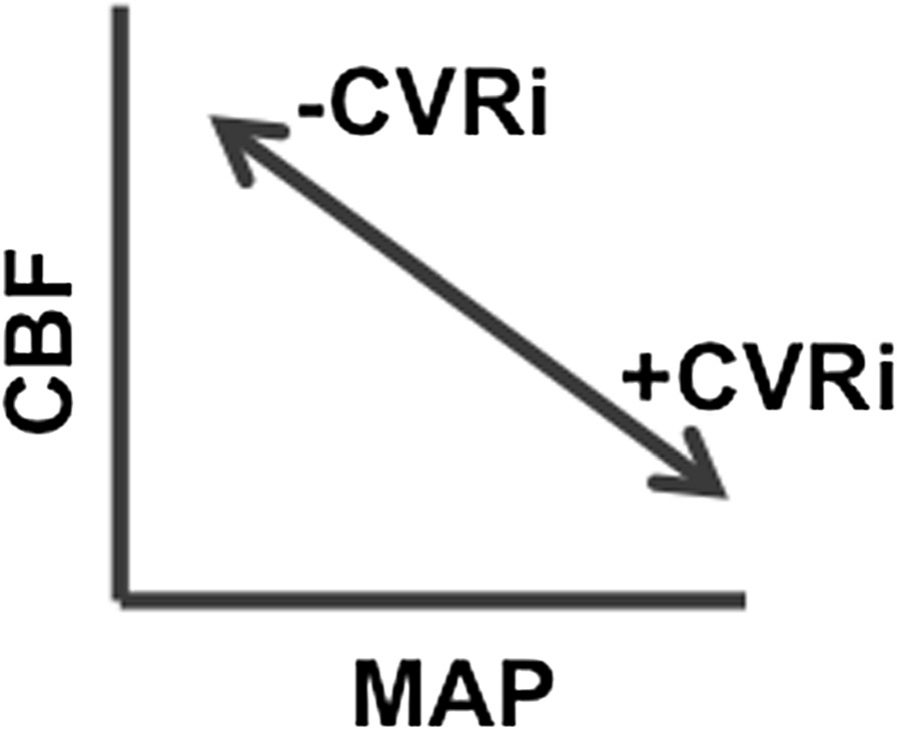
**Hypothesized relationships depicting elevation in CVRi in the very-old resulting from increased MAP and reduced CBF. CBF, cerebral blood flow; CVRi, index of cerebrovascular resistance; MAP, mean arterial pressure.**

In a recent study, we demonstrated that individuals with AD exhibit an elevated index of cerebrovascular resistance (CVRi) relative to non-demented older adults [20]. In the current study, we expand on these initial findings by examining the relationship between MAP, CBF, CVRi and cognition in a sample of non-demented older adults. Analyses compared young-old (ages 60 to 75) and very-old (80+) participants to identify clinically relevant differences in MAP, CBF and CVRi between these age groups. The aims of the current study included: 1) investigate the relationship between MAP and regional CBF in both age groups; 2) compare regional CVRi and CBF between the two age groups; and 3) examine if variations in CVRi were associated with cognitive performance. We hypothesized that there would be a negative relationship between MAP and CBF in both groups (for example, higher MAP associated with lower CBF), but that this relationship would be more pronounced in the very-old group due to typical increases in MAP and reductions in CBF with advancing age. Reflecting the expected higher MAP and lower CBF in the very-old, we hypothesized that the very-old group would, in turn, exhibit elevated CVRi compared to the young-old group (see Figure 1). Furthermore, we hypothesized that elevated CVRi would be associated with poorer cognitive performance, particularly within memory and executive functioning domains.

## Methods

### Participants

Participants were non-demented older adults participating in a longitudinal study of aging at the University of California, San Diego (UCSD) and the VA San Diego Healthcare System. All participants were independently functioning older adults recruited into the aging study from the San Diego, CA community via newspaper advertisements and flyers placed in senior centers targeting individuals who were either healthy, normally aging older adults or who were at-risk for developing AD. Participants were also recruited into the aging study from the UCSD Alzheimer’s Disease Research Center. A total of 58 participants were included in the current study. Thirty-eight of the participants were classified as young-old (between 60 and 75 years of age) and 20 participants were classified as very-old (80 years of age or older). The young-old and very-old groups were similar in terms of gender distribution and years of education. Exclusion criteria for the study included a current diagnosis of dementia, current or history of other major neurological disorders, such as Parkinson’s disease or stroke, history of a head injury involving a loss of consciousness, current or history of a serious mental illness or alcohol/substance abuse, and a pacemaker or other magnetic resonance imaging (MRI) contraindications. The study was approved by the institutional review boards at UCSD and the VA San Diego Healthcare System, and written informed consent was obtained from all participants.

### Data acquisition

MRI data were acquired on a GE Signa HDx 3-T whole body MR scanner using an 8-channel receive-only head coil. Structural images were acquired with high-resolution T1-weighted scans using a three-dimensional FSPGR sequence (25 cm FOV, 256 × 192 matrix TR = 7.8 msec, TE = min full, flip angle = 8°, inversion time = 600 ms, bandwidth = 31.25 kHz, and 172 1 mm sagittal slices; *n* = 49) or MP-RAGE sequence (identical parameters to FSPGR except 26 cm FOV, 256 × 256 matrix, TR = 7 msec, inversion time = 900 msec, 170 1.2 mm sagittal slices; *n* = 9). For a subset of 35 participants (13 very-old, 22 young-old), T2-weighted fluid attenuated inversion recovery (FLAIR) images were also acquired (20 cm FOV, 320 × 224 matrix, flip angle = 90°, TE = 124 ms, TR = 8,500 ms, 4 mm axial slices with no interslice gap).

Resting CBF images were acquired using pulsed arterial spin labeling (ASL) with a modified flow-sensitive alternating inversion recovery sequence [21] (post-saturation and inversion times of TI1 = 600 msec and TI2 = 1,600 msec, TR = 2.5 sec, TE = minimum, FOV = 22 × 22 cm, 64 × 64 matrix, 20 5 mm axial slices, 40 volumes (20 tag + control image pairs)). This sequence utilized presaturation pulses and QUIPSS 2 post-inversion saturating pulses and a spiral readout with four interleaves [22]. Additionally, a scan with the inversion pulses turned off was acquired to obtain an estimate of the magnetization of cerebrospinal fluid (CSF). The CSF signal was used to estimate the equilibrium magnetization of blood, which in turn was used to convert the perfusion signal into calibrated CBF units (ml/100 g/minute) [23]. A minimum contrast scan was also acquired to adjust for coil inhomogeneities during the CBF quantification step [24]. Each ASL dataset was reconstructed using the SENSE algorithm [25]. The ASL time series was co-registered to the middle time-point to minimize effects of participant motion. A mean ASL image was formed for each participant from the average difference of control and tag images using surround subtraction, and slice timing delays were accounted for to ensure the inversion time (TI2) was slice specific [26].

Two brachial artery BP recordings were obtained from each arm using an automated BP cuff while the participant was in a sitting position, and an average pressure from the two recordings was used. MAP was calculated using the following formula: diastolic BP + (pulse pressure/3), and pulse pressure (PP) was calculated by subtracting diastolic BP from systolic BP. APOE genotype was determined using a polymerase chain reaction-based method [10].

Memory functioning was assessed with the California Verbal Learning Test – 2^nd^ Edition (CVLT-II) [27] Long-Delay Free Recall z-score, executive functioning was measured with the Delis-Kaplan Executive Function System (D-KEFS) [28] Trail Making Test (Number-Letter Switching) scaled score, and language functioning was assessed with the D-KEFS Verbal Fluency (Letter Fluency) scaled score.

### Data processing

Data were processed using Analysis of Functional NeuroImages (AFNI; afni.nimh.nih.gov), FMRIB Software Library (FSL, Oxford, UK), and locally created Matlab scripts (The Mathworks Inc., Natick, MA, USA, 2010). Skull-stripping was completed using Brain Surface Extractor with manual editing to remove residual non-brain material [29] and images were segmented into gray matter (GM), white matter (WM) and CSF using FMRIB’s Automated Segmentation Tool. The high-resolution T1-weighted images were registered to ASL space and the partial volume segmentations were registered and down-sampled to the ASL data resolution. For quantification of white matter lesion (WML) volume, a semiautomated volumetric approach was applied to T2 FLAIR images using a previously described method that has been shown to be reliable [30]. Briefly, WMLs were manually traced in 17 to 21 image slices in the axial plane using AFNI. Total WML volume was quantified as the total voxel counts (mm^3^). Specific data processing procedures for ASL images have been previously reported [20]. Briefly, ASL data were converted to absolute units of CBF, corrected for partial volume effects [31], spatially smoothed using a 4.0 mm full-width, half-maximum Gaussian filter, normalized to Talairach space [32], re-sampled to a 4 × 4 × 4 mm resolution grid, and masked to include only supratentorial gray matter CBF.

CVRi was calculated by dividing MAP by CBF in each voxel (MAP/CBF). Secondary analyses were conducted using PP instead of MAP (PP/CBF). *A priori* regions of interest (ROIs) included two subcortical (caudate, thalamus) and two cortical (medial temporal lobe (MTL), posteromedial) regions defined using the Talairach atlas in AFNI. These regions of interest were chosen based on vulnerabilities to small vessel disease (subcortical) and AD pathology (cortical).

### Statistical analyses

All statistical tests used a significance cutoff of *P* < .05. All analyses were *a priori* unless specified as *post-hoc* analyses. Voxelwise analyses were conducted using AFNI software and all other analyses were conducted within SPSS software version 21.

### Demographic and clinical analyses

Independent samples *t*-tests (continuous variables) or chi-square (dichotomous variables) analyses compared age groups on demographic and clinical variables. Mann–Whitney U and Spearman correlational analyses were used to examine WML volumes due to non-normal distribution of the data.

### Relationships among age, MAP and CBF

#### ROI analyses

Pearson bivariate correlations were conducted to examine relationships among age, MAP and mean CBF within each of the four ROIs.

#### Voxelwise analyses

Voxelwise regression analyses examined the relationship between MAP and CBF across the whole brain. Type I error associated with multiple comparisons in voxelwise analyses was controlled based on Monte Carlo simulation results using AFNI’s AlphaSim program set with an individual voxel alpha of 0.05. Based on an averaged GM mask created from all participants, a minimum cluster volume threshold of 1,344 mm^3^ or 21 contiguous voxels was chosen and this threshold/volume combination protected a whole-brain probability of false positives of *P* < .05. Mean CBF values were extracted from significant clusters resulting from the voxelwise regression analysis, and these CBF values were included in *post-hoc* exploratory multiple linear regression models using a significance cutoff of *P* < .05. These *post-hoc* regression models included age group, MAP and an age group × MAP interaction term as predictor variables and mean CBF within each cluster as the outcome variable.

### Between-group differences in CBF and CVRi

Voxelwise two-tailed *t*-test analyses compared the young-old and very-old groups on whole brain CVRi and CBF. Additional *t*-test analyses also compared the two age groups on PP/CBF. Correction for multiple comparisons was completed using the procedure described above that protected a whole-brain probability of false positives of *P* < .05.

### Relationship between CVRi and cognition

Hierarchical multiple linear regression analyses tested hypothesized interaction effects between age group and CVRi within each ROI on measures of memory, executive functioning and language. Each model included gender and education as covariates in the first block, the main effects of age group and CVRi in the second block, and the interaction of age group and CVRi in the third block. Standardized scores on each cognitive measure were used as outcome variables.

## Results

### Demographic and clinical characteristics

The young-old and very-old groups did not differ in gender distribution (*P* = .55), years of education completed (*P* = .69) or distribution of APOE ε4 carriers (*P* = .39) (see Table 1). The very-old group exhibited significantly elevated PP (*P* < .05) compared to the young-old group, but the groups did not significantly differ on MAP (*P* = .06). Additionally, the very-old group did not significantly differ from the young-old group in anti-hypertensive medication use (*P* = .07). The two age groups also demonstrated similar performances on the three neuropsychological measures included in this study (*P*s > .42). Table 1 displays the means and standard deviations of the demographic and clinical variables for each age group.

**Table 1:** **Demographic and clinical characteristics of young-old and very-old groups**

	**Young-old (60 to 75)**	**Very-old (80+)**	***P***
Number	38	20	
Age (years)	69.1 (3.9)	84.8 (4.0)	<.001
Gender (M/F) (number)	14/24	9/11	.55
Education (years)	15.9 (1.9)	15.7 (3.0)	.69
APOE (ε4-negative/ε4-positive) (number)	21/15	14/6	.39
Systolic BP	125.8 (14.2)	136.2 (17.8)	<.05
Diastolic BP	74.9 (7.7)	77.8 (11.7)	.26
Mean arterial pressure	91.9 (8.6)	97.3 (13.0)	.06
Pulse pressure	50.9 (12.4)	58.4 (11.3)	<.05
Anti-hyperintensive medication use (number currently prescribed medication/*n*umber not currently prescribed medication)	17/21	14/6	.07
CVLT-II Long delay free recall *z*-score	0.3	0.6	.42
D-KEFS Verbal fluency (letter) ss	12.4	13.2	.46
D-KEFS Trail making test switching ss	12.3	12.1	.82

Means (standard deviations); BP, blood pressure; CVLT-II, California Verbal Learning Test – 2^nd^ Edition; D-KEFS, Delis-Kaplan Executive Function System; M/F, male/female; ss, scaled score.

### Relationships among age, mean arterial pressure and cerebral blood flow

Across the total sample, age was positively correlated with MAP (*r* = .33, *P* = .01). There was a negative relationship between age and CBF in the thalamus (*r* = −.24, *P* < .05), but non-significant correlations between age and CBF in the remaining ROIs (*P*s > .20). MAP was not correlated with mean global CBF (*r* = −.13; *P* = .34).

**Figure 2:**
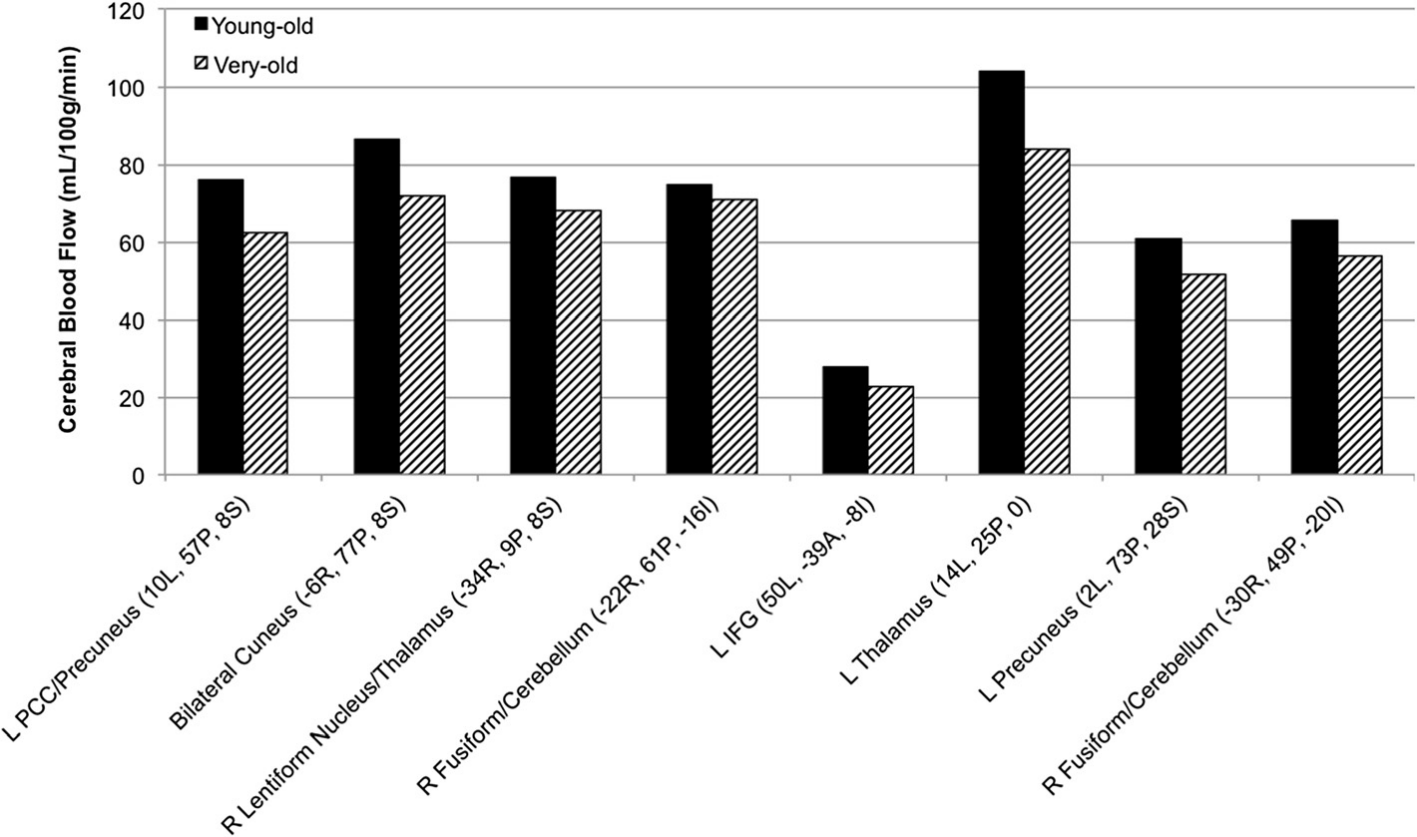
**Mean CBF for young-old (black) and very-old (striped) groups within each significant cluster.** Clusters shown resulted from voxelwise regression of MAP on CBF and survived the cluster threshold alpha-protection procedure (*P* < .05, volume >1,344 mm^3^). Coordinates (x, y, z) refer to maximum intensity values within each cluster (L = left, R = right, A = anterior, P = posterior, S = superior, I = inferior). CBF, cerebral blood flow; MAP, mean arterial pressure.

Voxelwise regression analyses revealed negative relationships between MAP and regional CBF in posterior regions (posterior cingulate, precuneus, cuneus, fusiform gyrus), subcortical regions (thalamus, lentiform nucleus) and inferior frontal gyrus across the total sample. Exploratory *post-hoc* analyses examined whether the relationship between MAP and CBF from within each significant cluster differed between the young-old and very-old groups. Figure 2 displays the mean CBF values for each age group within each significant cluster. These *post-hoc* analyses revealed significant main effects of MAP in the five posterior clusters (all *P*s < .05), indicating a negative relationship between MAP and CBF across both age groups. A significant interaction effect between age group and MAP on CBF was observed in the thalamus (*P* < .05). Simple effects analyses revealed that MAP accounted for a significant amount of variance in thalamus CBF in the young-old group (*β* = −2.47; *P* < .001), but was non-significant in the very-old group (*β* = −.78; *P* = .17).

### CVRi differences between age groups across the whole brain

**Figure 3:**
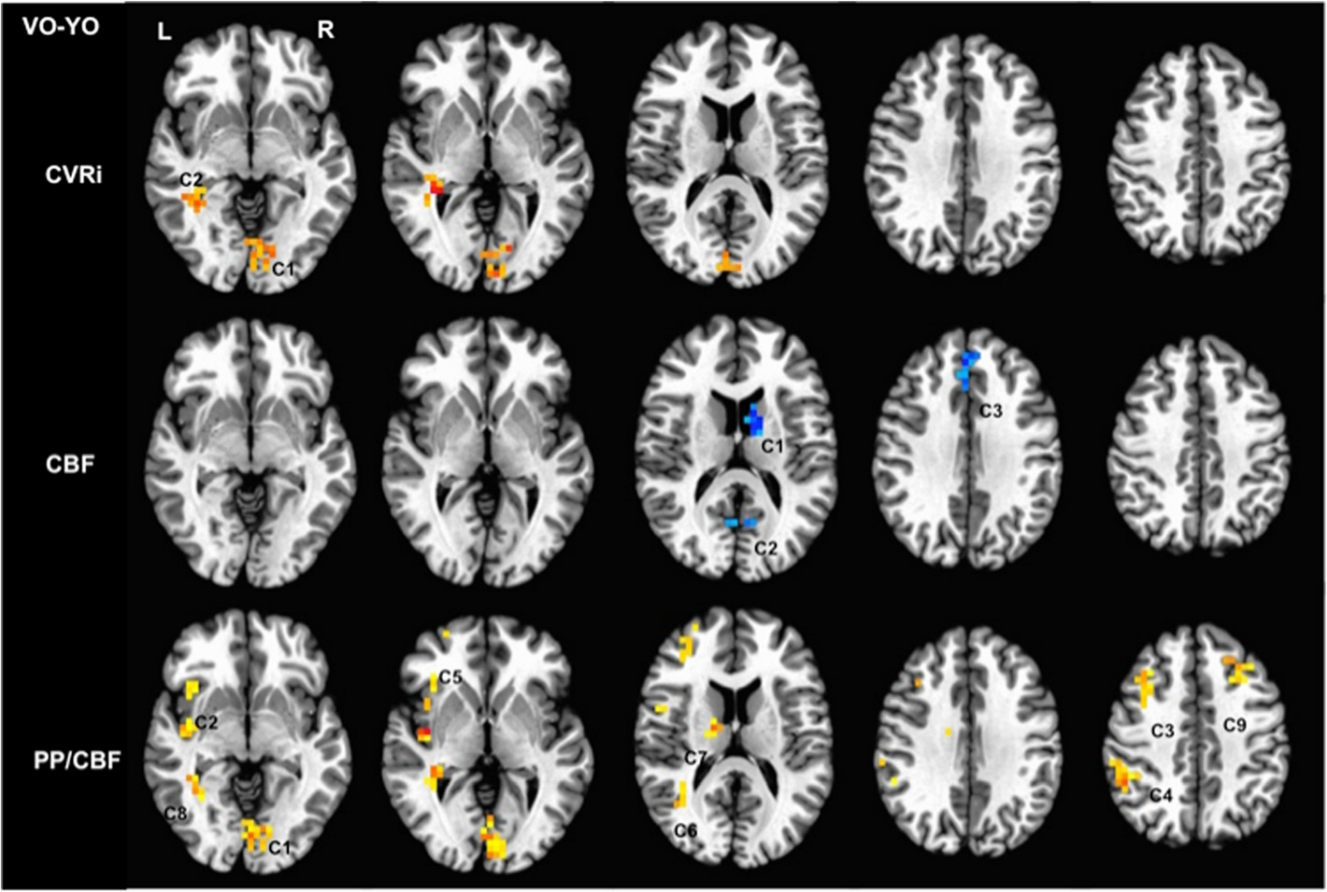
**Significant clusters of reduced CBF and elevated CVRi and PP/CBF indices in the very-old group.** Significant differences at corrected *P* < .05. CBF, cerebral blood flow; CVRi, cerebrovascular resistance index; PP/CBF, ratio of pulse pressure to cerebral blood flow.

Voxelwise *t*-test analyses resulted in two significant clusters in the parahippocampal gyrus/caudate and cuneus/lingual gyrus in which the very-old group exhibited significantly elevated CVRi compared to the young-old group (see Table 2). Reduced CBF in the very-old group relative to the young-old group was exhibited in the caudate, medial frontal gyrus, and precuneus. *Post-hoc* analyses demonstrated PP/CBF elevations in the very-old group in similar regions as CVRi, as well as additional anterior regions, parietal regions, middle temporal gyrus and thalamus. Group differences in CVRi, PP/CBF and CBF are displayed in Figure 3.

**Table 2:** **Significant clusters resulting from voxelwise** ***t*** **-tests comparing CVRi and CBF between young-old and very-old groups**

**Region**	**Volume (mm** ^**3**^ **)**	**Maximum intensity (x, y, z)**	**Maximum intensity** ***t***
**CVRi**			
(1) L/R Cuneus/lingual gyrus	3136	6 L, 69P, −8I	2.46
(2) R Paraphippocampal gyrus/superior temporal gyrus/caudate	1536	−42R, 41P, −20I	2.08
**CBF**			
(1) L Caudate/lentiform nucleus	1600	14 L, 1P, 8S	−2.38
(2) L/R Posterior cingulate, L/R cuneus, L precuneus	1280	−6R, 61P, 8S	−2.57
(3) L/R Medial frontal gyrus	1280	2 L, −43A, 24S	−3.04
**PP/CBF**			
(1) L/R Lingual gyrus/cuneus	3136	14 L 69P -12I	2.05
(2) R Insula/inferior frontal gyrus	2816	−42R 5P -8I	4.66
(3) R Middle frontal gyrus/R superior frontal gyrus	2112	−34R -23A 32S	2.94
(4) R Inferior parietal lobule/supramarginal gyrus	1920	−58R 29P 32S	2.60
(5) R Superior frontal gyrus/R middle frontal gyrus	1664	−26R -55A 0	2.37
(6) R Middle temporal gyrus	1664	−42R 49P 8S	2.42
(7) R Thalamus/caudate/cingulate gyrus	1472	−14R 5P 12S	2.00
(8) R Fusiform gyrus/paraphippcampal gyrus/caudate	1344	−42R 41P -20S	2.41
(9) L Middle frontal gyrus	1280	22 L -31A 36S	2.28

Clusters shown survived our cluster threshold alpha-protection procedure (*P* < .05, volume >1,280 mm^3^). A, anterior; CBF, cerebral blood flow: CVRi, index of cerebrovascular resistance; I, inferior; L, left; P, posterior; R, right; PP, pulse pressure; S, superior.

### Relationships between regional CVRi and total WML volume

Mann Whitney U tests revealed that the very-old group exhibited greater total WML volume *(P* = .001*)*, periventricular WML volume (*P* = .001) and deep WML volume (*P* < .05) compared to the young-old group. Additionally, there was a positive association between periventricular WML volume and CVRi in the caudate (*rs* = .35; *P* < .05).

### Interaction between age group and CVRi on cognitive performance

**Figure 4:**
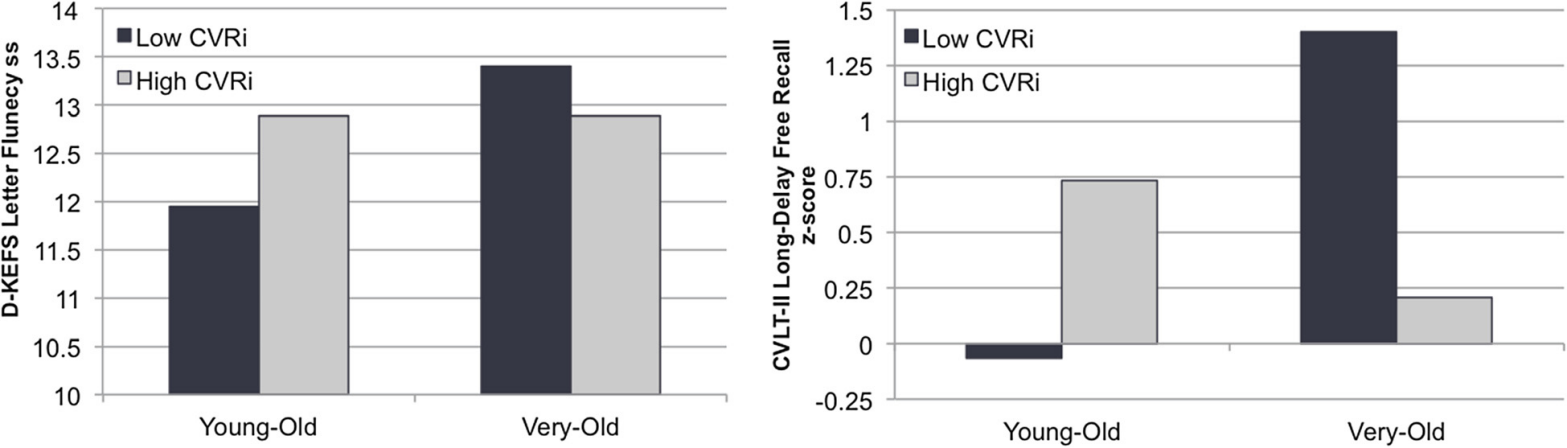
**Mean cognitive scores for participants with above average (black) and below average (gray) CVRi.** CVRi, index of cerebrovascular resistance; ss, scaled score; CVLT-II California Verbal Learning Test – 2nd Edition; D-KEFS, Delis-Kaplan Executive Function System.

Hierarchical linear regression analyses resulted in a significant interaction effect between age group and thalamus CVRi on language (verbal fluency) performance (Δ*R*^2^ = .06, *t* = −2.03, *P* < .05). Follow-up simple main effects analyses demonstrated a negative relationship between thalamus CVRi and verbal fluency performance in the very-old group, whereas there was a positive relationship between CVRi and verbal fluency performance in the young-old group. Additionally, there was a non-significant trend towards an interaction between age group and MTL CVRi on memory (word recall) performance (Δ*R*^2^ = .07, *t* = −1.91, *P* = .06). Follow-up simple main effects analyses depicted a negative relationship between memory function and CVRi in the MTL in the very-old group, but a positive relationship in the young-old group. Figure 4 displays mean performance on verbal fluency and verbal memory measures for those with lower than average CVRi and higher than average CVRi within each age group. There were no significant interaction effects between age and CVRi on executive functioning (for example, set-shifting).

## Discussion

This study investigated the relationship between MAP and CBF in older adults of varying ages. In a sample of non-demented older adults, higher MAP was associated with reduced CBF in subcortical regions, posterior cortical regions and inferior frontal gyrus. This negative relationship was similar in young-old (ages 60 to 75) and very-old groups (age 80+) suggesting that functions of the microvasculature within these regions may be vulnerable to elevated BP effects in older adults across the aging spectrum. For example, higher baseline systolic and diastolic BP in older adults has been reported to be significantly associated with reduced total CBF over a four year follow-up period [33]. The relationship between MAP and our measure of regional microvascular CBF (ASL-MRI) was observed despite the lack of a significant correlation between MAP and global CBF, suggesting that global or large vessel CBF measures may be less sensitive to subtle or regionally specific cerebrovascular dysfunction associated with age-related BP elevation.

Despite a similar relationship between MAP and CBF across age groups, the very-old group exhibited elevated CVRi in the caudate, parahippocampal gyrus and cuneus. An alternate estimate of CVRi using pulse pressure rather than mean arterial pressure (PP/CBF) also exhibited elevations in the very-old group in similar regions as our original CVRi estimate, as well as in additional anterior, parietal and temporal regions. The greater number of regions implicated with the use of PP compared to MAP in our CVRi estimate may be due to the larger elevation in PP versus MAP observed in our sample. Elevated CVRis have previously been reported in individuals with AD and mild cognitive impairment [14,20], and our current findings suggest that regional cerebrovascular resistance elevations also occur in advanced age. The particular regions that demonstrated CVRi elevations in the very-old have been shown to be vulnerable to cerebral small vessel disease (for example, caudate) or neurodegeneration in early stages of AD (for example, parahippocampal gyrus, cuneus).

Moreover, prior studies have implicated the role of cerebral hemodynamic dysfunction in elevated WM disease [34]. Similar to previous studies, the very-old group exhibited greater WML burden compared to their younger counterparts [35]. Furthermore, elevated CVRi in the caudate, a subcortical region sensitive to small vessel disease due to perfusion by small terminal arteries with little collateral flow [36], was associated with greater WML volume. Evidence suggests that elevated BP, as well as fluctuating BP over time, is associated with elevated WML burden [19], and that reduced CBF occurs in regions with WML [37]. Therefore, elevated subcortical CVRi may be a potential marker for age-related cerebrovascular dysfunction associated with cerebral small vessel disease.

Furthermore, elevated thalamic CVRi was associated with poorer verbal fluency performance within the very-old group, whereas CVRi was not associated with cognition in the young-old group. A similar, though non-significant (*P* = .06) pattern was observed between elevated MTL CVRi and poorer verbal memory performance in the very-old group. These results suggest that elevated BP in the context of reduced CBF may have a more detrimental impact on cognitive functioning in advanced age and has regionally specific effects on cognitive abilities subsumed by those regions.

Further studies are needed to investigate the etiology of elevated cerebrovascular resistance in the very-old. One hypothesis of elevated cerebrovascular resistance in older adults with AD that has been proposed is vasoconstriction associated with increased amyloidosis [14]. Although the participants in this study were non-demented, it is possible that elevated cerebrovascular resistance in the very-old may also reflect underlying amyloidosis, consistent with greater Alzheimer’s pathology often observed in the oldest-old at autopsy. Additionally, recent animal studies demonstrate that aged hypertensive mice exhibit cerebral autoregulatory dysfunction, and that this dysfunction is associated with cerebromicrovascular damage, including disruption of the blood–brain barrier and neuroinflammation [19]. It is possible that elevated CVRi in the very-old may reflect capillary dysfunction that contributes to neurovascular dysfunction and increases risk for development of AD pathology and cognitive decline [38]; however, these hypotheses will need to be investigated further in future studies.

There are several limitations of the current study that should be considered. First, our sample size was relatively small, particularly that of the very-old group, and it will be important to validate the current findings and explore the relationship between BP and CBF in a larger sample of very-old participants in the future. Additionally, our measure of arterial BP was conducted outside of the MRI scanner, rather than concurrently acquired with the CBF data. Future studies using an MRI-compatible BP monitor may be able to calculate a more direct index of regional cerebrovascular resistance within particular structures, which may be more sensitive to these age-related changes in pressure-flow relationships within the brain. Additionally, several analyses were conducted in this study and, although we corrected for multiple comparisons in our voxelwise analyses, it is possible that some of our findings may be inflated due to Type I error. As noted above, additional follow-up studies to explore and validate these findings in separate samples will be needed to confirm the accuracy of these results. Additionally, due to the cross-sectional nature of the study we are unable to determine causal relationships between elevated CVRi and its regional associations with cognitive function. However, these participants will continue to be followed annually and future studies will provide information regarding cognitive change in these individuals. Longitudinal follow-up studies with neuropathologic data will also be helpful in validating the relationship between CVRi elevations and occult cerebrovascular disease at autopsy. Lastly, although the young-old and very-old participants in this study had similar patterns of anti-hypertensive medication use, we did not have access to detailed information regarding the duration of medication use within our two groups. Further investigation of the contribution of anti-hypertensive medication use on CBF and CVRi in the very-old will be important to assess in future studies.

## Conclusions

Our study provides evidence that an index of cerebrovascular resistance is elevated in a non-demented very-old group within regions associated with AD and small vessel disease, and that elevated CVRi is associated with regionally specific effects on cognitive abilities subsumed by those regions in the very-old. Our results suggest that continued investigation of CVRi may be warranted as it may represent a useful marker for occult cerebrovascular disease related to vascular aging. Previous studies indicate that cognitive and morphometric changes in very-old AD patients are less salient than those in young-old patients; therefore, additional markers may be needed to enhance the ability to detect early AD in individuals at the upper end of the age spectrum [39].

## Abbreviations

AD: Alzheimer’s disease
AFNI: Analysis of Functional NeuroImages
APOE: apolipoprotein
ASL: arterial spin labeling
BP: blood pressure
CBF: cerebral blood flow
CSF: cerebrospinal fluid
CVLT-II: California Verbal Learning Test – 2^nd^ Edition
CVRi: cerebrovascular resistance index
D-KEFS: Delis-Kaplan Executive Function System
GM: gray matter
ICP: intracranial pressure
MAP: mean arterial pressure
MRI: magnetic resonance imaging
MTL: medial temporal lobe
PP: pulse pressure
P_*α*_: cerebral perfusion pressure
ROI: region of interest
WM: white matter
WML: white matter lesion
